# From Tutoring Gross Anatomy to Pancreatic Surgery Innovation

**DOI:** 10.3390/ijerph19010359

**Published:** 2021-12-30

**Authors:** Alberto Balduzzi, Giovanni Marchegiani

**Affiliations:** Department of General and Pancreatic Surgery, The Pancreas Institute, University of Verona Hospital Trust, 37134 Verona, VR, Italy; alberto.balduzzi@univr.it

**Keywords:** anatomy, gross anatomy, pancreas, pancreatic surgery, minimally invasive surgery, anatomy dissection

## Abstract

The training for pancreatic surgery still is not conducted according to standardized protocols, and academic programs differ between countries and hospitals. Moreover, due to recent technological innovations such as minimally invasive and robotic surgery, and the broader indications for complex pancreatic procedures due to the use of neoadjuvant chemotherapy, training is continuously redefining itself. The historical paradigm of “see one, do one, teach one” has been challenged and might have become obsolete. Finally, the rising number of surgical residents along with the limited time required practicing during residency might represent a major limitation to becoming an independent surgeon. Gross anatomy is a solid practice for the active learning of human anatomy during medical school. With regards to the pancreas, it offers a unique opportunity to both actively study the pancreatic gland anatomy during dissection and simulate actual surgical procedures. A critical review of the literature was conducted, aiming to assess the role of gross anatomy in surgical training and possible future perspectives.

## 1. Introduction

Acknowledged as a fundamental component of medical education, human anatomy offers a wide range of teaching resources and strategies. However, the current medical school curriculum has deeply changed due to the expansion of biomedical knowledge along with the rapid evolution of medical practice. The time dedicated to teaching gross anatomy to undergraduate medical students is rapidly decreasing. Available data show a negative trend of gross anatomy hours in students’ curricula from a mean of 248.7 h in 1973 down to 143.6 h in 2001 [1] and 129 h in 2017 [2]. These results underline the risk that post-graduate students might lack proper anatomical preparation for residency. As a direct consequence of this, anatomical knowledge is lacking in those residency programs where it is most essential, namely, the surgical ones. Concerns regarding the anatomical preparation of medical school graduates before starting residency programs were underlined back in 1999 by Cottam et al. [3] Program directors admit that less than half of incoming residents show adequate anatomical knowledge and the anatomical knowledge of 14% seriously lacking. Interestingly, most students recognize anatomy as “very” to “extremely” important to medical training. However, they believe their anatomy knowledge to be insufficient [4].

In recent times, new data have shown that anatomy knowledge is often called into question during litigation regarding surgical malpractice. In the United Kingdom, the Medical Defense Union received a seven-fold increase in claims associated with anatomical incompetence, as observed by Ellis et al. [5].

According to Brenner et al. [6], anatomy-teaching tools can be divided into six categories: dissection by students, inspection of prosected specimens, didactic teaching, use of models, computer-based learning (CBL), and teaching living and radiological anatomy. These tools aim to embrace all the possible modalities of the available teaching models for medical students. However, notable changes over time regarding both the anatomy curriculum and students’ knowledge of human anatomy questioned the current application and use of teaching tools.

Another relevant point is the context in which anatomy is taught. A clinically meaningful context with clear reference to the competences required by medical graduates is a pivotal point for courses intending to impress the relevance of anatomy on medical students for their future practice [7].

The pancreas represents a surgical challenge due to the anatomical location of the gland in the retroperitoneum, and the multivisceral relations with important structures and major blood vessels. Pancreatic surgery requires targeted anatomical knowledge, along with high-level surgical skills, and awareness of possible postoperative surgical complications. Indeed, postoperative sequelae, such as postoperative pancreatic fistula, might result in a longer hospital stay, worsening of patients’ clinical conditions, reintervention, or even death. The rising incidence of pancreatic diseases implies the need for the implementation of training for surgeons approaching this highly specialized surgical field [8]. In recent years, pancreatic surgery has been subject to increasing innovations regarding both the management of complications and surgical strategies. Notwithstanding, the surgical load of practice required by pancreatic surgery, anatomical knowledge and preoperative evaluation of possible anatomical variation is always an essential basis to guarantee patient safety. 

In the present review, we aim to critically present the current issues regarding tutoring on the gross anatomy and underline future perspectives, with a focus on pancreatic surgery.

## 2. Materials and Methods

We have conducted a critical review of the literature focused on the current medical curricula, anatomical learning tools, and development of surgical skills integrated with human anatomy, aiming to provide a comprehensive overview of the current status and possible future perspectives, emphasizing pancreatic surgery.

## 3. Results

### 3.1. Diversity and Integrated Curricula

The reduction in the anatomy curriculum is mainly due to the introduction of new disciplines to undergraduate teaching [9]. To provide a standard for safe and effective clinical practice, a syllabus has been developed, together with reforms aiming to establish vertically integrated curricula [10]. This model was meant to improve anatomical knowledge among newly qualified physicians. The introduction of clinical sciences in early years, with continuative attention to anatomy and basic sciences in later years, allowed different areas of anatomical knowledge that should be prioritized [11,12]. Knowledge of these areas should be gained and improved over the course of study according to the clinical experience of the student. A vertically integrated approach was proven to be effective in settling the anatomical knowledge applied to clinical practice [13,14]. Therefore, students feel motivated in learning both basic sciences and clinical medicine, acquiring a holistic approach for future clinical practice [14,15]. The downside of the vertical integration of anatomy across both clinical and pre-clinical years is the risk of being “unidirectional”. While clinical sciences can easily be integrated and anticipated during pre-clinical years, this cannot easily be applied to the basic sciences during clinical years [11]. Therefore, a strong effort from faculty members and administrations is crucial to guarantee the necessary coordination between departments. In the absence of these initiatives, students risk not obtaining an overall picture of human anatomy, which is an essential tool for a safe clinical practice [11,16].

### 3.2. Gross Anatomy, Cadaver Dissection, Prosection, and Plastination

Over the years, anatomical dissection has proven to be an effective teaching tool for medical students. The advantages of gross anatomy are represented by working and practicing on the human body with a deeper understanding of anatomical structures and relations. Along with the theoretical knowledge, which is mandatory for medical students, anatomical dissection could help develop other qualities that, as physicians, should eventually be applied in future clinical practice. Dissection provides practice and manual skills, allows for an understanding of the possible relationships between symptoms and pathology, and prepares students to encounter death [17,18]. Moreover, as underlined by Bockers et al. [19], it plays an important role in building medical professionalism, teamwork competencies, stress coping strategies, and empathy with the working group. Therefore, one could say that cadaver dissection is crucial to forge future physicians [20]. Given the compelling role of gross anatomy, many anatomists believe that—when possible—it should be chosen over other teaching tools [21]. Even if cadaver dissection is recognized as an essential tool, it could be inadequate for modern undergraduate students [20]. Moreover, cadaver dissection has high costs and must requires a tremendous amount of time [22]. A crowded curricula, low number of mortuaries, high maintenance costs, health concerns for chronic exposure to chemicals (formalin fumes), and ethical and medico-legal issues are threatening cadaver dissection [23,24,25]. A solution could be represented by cadaveric prosections, namely, already dissected anatomical specimens. It has been observed that prosections provide a system-based, less time-consuming approach that does not require anatomical dissection and preparation [26]. In this setting, prosection-based courses have proven to be time-efficient and flexible with fewer cadavers being needed, since a higher number of students can benefit from the same preparation for multiple sessions [27,28]. Another advantage compared to cadaveric dissection is that students can observe and study a higher number of anatomical variations using prosections. However, the advantages of prosections over cadaver dissections have yet to be proven [29].

The preservation of anatomical dissection can be obtained using formalin or plastination; however, both techniques have limitations. First described in 1977 [30], plastination allows for the fixation of anatomical specimens over a long period of time (almost 10 years) with low storage costs and no need for flume extraction [31]. The disadvantages of plastination are the shrinkage of tissues, and loss of texture and fine details [30].

### 3.3. Innovations and Modern Integrations in Anatomical Learning Tools

Radiological imaging, including high-resolution computed axial tomography (HR-CAT), magnetic resonance imaging (MRI), and ultrasound (US), allow for the display of high-resolution images, showing both radiological anatomy and pathological processes, in 2D and 3D [32]. When applied to the anatomy curricula, radiological imaging allows for the integration of anatomical knowledge with clinical scenarios. Moreover, the primary drawbacks of dissection, according to students, are its time-consuming nature and the difficulty in accurately recognizing anatomical structures [33]. However, students’ perceptions of the challenges connected with dissection in recent years are likely to facilitate critical thinking and stimulate the development of professional skills that can assist in overcoming hurdles and addressing issues as clinicians [34].

### 3.4. From Gross Anatomy to Clinical and Surgical Skills

According to researchers, medical students establish emotional attachments to donated cadavers while dissecting them, which later helps them understand the psychological elements connected with the patient’s sickness and contributes to the development of effective future physicians. Body donation programs are being used all around the world to help medical students develop humanistic characteristics such as respect, understanding, and compassion [35,36,37,38,39]. Analyzing the potential stress derived from cadaver dissection, authors have stated that dissection helps students to learn to adjust their emotional reactions and attitudes, establishing cadaveric dissection as a valuable instructional method and an excellent tool for medical students to integrate professional and technical abilities [40,41]. Most students see dissection as the finest technique to study anatomy and believe it is essential to anatomical sciences [42]. Few students would genuinely choose cadaver dissection above other accessible teaching/learning methods because they believe that the dissected cadaver provides the finest possible 3D comprehension of human anatomy [43]. They believe that dissection stimulated their senses of sight and touch, resulting in a better grasp of anatomy. When students are called upon to evaluate patients as future practicing physicians, they must be able to appreciate visual and tactile cues [44]. Learning anatomy through dissection enables students to link and correlate information, allowing them to develop conceptual insights that are essential for deep and rewarding learning [45]. Students have also stated that studying via dissection provides them with a foundation that will help them become better doctors in the future. This is important in the medical curriculum because a greater grasp of fundamental science is required to link scientific principles to practical clinical situations, which is required for the development of cognitive abilities [44]. Furthermore, medical students have emphasized the sanctity of the cadaver as a human specimen. They have conveyed their sorrow and respect while acknowledging that cadavers were once living beings [42]. As a result, it is possible that the corpse, as the first teacher in medicine, instills a sense of care, warmth, and empathy in students, all of which are crucial for future medical practice.

### 3.5. Improving Surgical Skills through Gross Anatomy

Cadaver dissection remains the most effective way of presenting and learning anatomy as a dynamic basis for problem-solving. However, the current trend of a gradual reduction in dissection practice and, as a result, inadequate anatomical knowledge is a source of worry not just for undergraduates, but also for postgraduates in surgical practice specialties [46]. Anatomy learning based on dissection is important for surgeons, but it is also useful for any clinician who performs invasive procedures on patients. It provides a knowledge base that applies to all medical vocations [47]. Dissection, according to medical specialists at all levels, aids in the development of practical skills. Furthermore, they believe that dissection-based anatomy teaching is linked to clearly defined and attainable goals, allowing students to ingest anatomy information with relevant clinical correlations [48,49]. Dissection training, according to surgeons, not only provides valuable insights into anatomical details but also familiarizes students with anatomical variations and an appreciation of fully exposed structures, which, while not visible in the operating room, are at risk of being inadvertently damaged during surgery [47]. Significant gains in written and oral examination scores, operative confidence, and video assessments of operating techniques have been documented in studies evaluating the efficacy of cadaveric surgery among surgical trainees [50,51,52]. This style of dissection was generally regarded to be entertaining and favorable to active thought by participants [52]. Several cadaveric surgery courses are available, but are mostly oriented in favor of surgical residents rather than undergraduate medical students [50,51,53]. On a lesser scale, cadaveric surgery has been carried out by integrating procedure-based dissections into a more typical head-to-toe dissection manual [54]. Inadequate anatomy knowledge has been highlighted as a key factor in graduating surgery residents’ lack of operating skills and confidence [55,56,57]. Evidence show that intraoperative mistakes cause two-thirds of morbidity and death in surgical patients, the majority of which are technical in origin [58]. Cadaveric procedural anatomy courses have been shown to improve the residents’ confidence and competence across a wide range of surgical procedures, with a consequent possible impact on patients’ safety [51]. The transition of chief residents to independently practicing surgeons requires a high level of operational autonomy and independence in the operating theatre. Changes in supervisory standards, work hour adjustments, and public conceptions of resident responsibilities in patient care have all hampered the development of these abilities throughout residency [59]. Moreover, based on rotation distribution and patient presentation, the diversity and unpredictability of general surgery training in a large volume facility may also alter a trainee’s exposure to different case types. The constancy of exposure to some operations throughout the early years of training is restricted. However, equipping general surgery residents with the essential abilities of managing differences and complexity that are experienced throughout an operation is a problem [50].

### 3.6. Current Advancements in Surgical Training for Pancreatic Surgery

Along with liver and biliary tree, pancreatic surgery was first considered to be within the domain of general surgeons. However, at present, pancreatic procedures are mostly performed at high-volume centers by fellowship-trained surgeons [60]. The increasing procedural volume and service line organization are crucial to high-quality postoperative results. Patient safety and postoperative outcomes have been connected to surgical expertise as well as the hospital environment’s ability to “rescue” patients from any possible issues [61,62,63]. Surgical volume, experience, and competence all influence the quality of postoperative outcomes [64]. Therefore, surgeons’ training is crucial to ensure quality outcomes for these patients. However, the hepato-pancreato-biliary (HPB) caseload during the general surgery residency program might not be adequate to train future surgeons to such a high-quality standard [65]. In a recent publication, Diaz et al. [65] underline that most general surgery residents perform fewer than 10 liver, pancreas, or complex biliary operations before graduating, and there is a widening heterogeneity among residents. As a direct consequence, most graduated general surgeons do not feel confident performing liver, biliary, and pancreatic procedures due to a lack of experience and training during residency [66]. Aiming to achieve such confidence, most newly graduated residents seek an HPB fellowship after graduation [67].

However, university centers have the responsibility to offer training for future generations of surgeons, in addition to maintaining high standards for patient care, research, and medical advancements. Residents in general surgery are exposed to a wide range of surgical techniques across the world. The tendency toward subspecialization in general surgery, notably in the difficult field of HPB operations, does restrict the resident burden in this area. A pressing concern is represented by the safety and suitability of general surgery trainees conducting complicated surgical operations while maintaining high surgical and oncological outcomes [8]. The published evidence in this regard is limited and the data are heterogeneous [68]. A recent study by Salvia et al. [8] shows that the selected major pancreatic surgeries can be safely performed by residents, with outcomes that are comparable to those of attending surgeons. Within the setting of supervised surgery, pancreatic resections are a viable teaching paradigm for surgical residents. From these results, it is possible to hypothesize further benefits deriving from a gross-anatomy course focused on pancreatic dissections and surgical procedures’ simulations.

## 4. Discussion

The relatively short time spent teaching anatomy to medical students represents an acknowledged issue in anatomy courses in recent years. However, many areas of interest are competing for a place in the medical curricula. Therefore, we should focus on improving the methods of teaching and learning human anatomy, instead of spending more time on a single course. Human anatomy and gross-anatomy courses have been shown to be crucial for both students and surgical residents. Moreover, there are many possible paths for teaching anatomy that would not add to the current curricula. One such path is represented by the vertical integrated curricula [10] aiming to improve anatomical knowledge among newly qualified physicians whose exposure to anatomy during training may be lacking. Indeed, combining several pedagogical methods that complement one another could result in a successful strategy for current courses. When multimodal and system-based techniques are used, students appear to learn more effectively, especially when applying the knowledge to clinical practice [14]. An integrated curricula could balance the gap between anatomical studies and clinical practice. During residency, anatomical knowledge is assumed to be part of the students’ educational background. An integrated curricula should also be considered for surgical residencies. In fact, the time devoted to clinical practice tends to shift the trainees’ attention away from the theory itself.

Surgery departments require trainees to highlight qualities that should be gained at the beginning of their study course. Professionalism, teamwork competencies, empathy with the patients and the working group, and stress coping strategies can be provided during gross-anatomy courses [17,18]. This is even more relevant for pancreatic surgery. Indeed, within this highly specialized surgery, gross anatomy contributes to the ability to face long and stressful surgeries, binding clinical hours, and challenging postoperative sequelae for the patients. However, there is currently no scientific evidence relating to the potential benefits of anatomical dissection during training in pancreatic surgery.

The authors had the opportunity to actively study human cadavers, starting from medical school, by taking part in anatomical dissections. This experience helped them obtain a better understanding of anatomy, organs, and vascular relations, but also confirmed their deep love for HPB surgery and the true values of working side-by-side with invaluable teachers and esteemed colleagues. Gross anatomy should be integrated into medical curricula throughout all the educational paths, from students to formal physicians, since it allows for a holistic approach to anatomy. In recent years, pancreatic surgery has significantly evolved thanks to the introduction of minimally invasive surgery (MIS). However, MIS training requires surgery to be performed as the first operator, limiting the chances of these techniques being learned during residency, whereas classical surgery allows for learning by the observation and imitation of surgical gestures. Adding gross-anatomy classes during surgical residency would enable young surgeons to simulate minimally invasive surgical procedures, abating their learning curve, especially if combined with radiological imaging applied to clinical scenarios.

## 5. Conclusions

In conclusion, the advantages of anatomical dissection underlined in the present manuscript underscore the potential that this would have in the training of forthcoming surgeons. Future pancreatic surgery training programs should be implemented, incorporating cadaver dissection and pancreatic surgical procedures in surgical residents’ curricula.

## Data Availability

Not applicable.
